# Immune Checkpoint Inhibitor-induced Hepatitis, an Emerging Issue in Precision Cancer Therapy Era: A Narrative Literature Review

**DOI:** 10.5041/RMMJ.10571

**Published:** 2026-01-28

**Authors:** Randy Adiwinata, Caroline Tanadi, Fegita Beatrix Pajala, Kevin Tandarto, Maureen Miracle Stella, Jeffry Beta Tenggara, Ralph Girson Gunarsa, Paulus Simadibrata, Lianda Siregar, Saut Horas Hatoguan Nababan, Budiman Sujatmika Sulaiman, Irsan Hasan, Cosmas Rinaldi Adithya Lesmana, Aru Wisaksono Sudoyo

**Affiliations:** 1 Gastrointestinal Cancer Center, MRCCC Siloam Hospital Semanggi, Jakarta, Indonesia; 2 School of Medicine and Health Sciences, Atma Jaya Catholic University of Indonesia, Jakarta, Indonesia; 3 Division of Hematology and Medical Oncology, MRCCC Siloam Hospital Semanggi, Jakarta, Indonesia; 4 Division of Gastroenterology and Hepatology, Department of Internal Medicine, Dharmais Cancer Hospital, Jakarta, Indonesia; 5 Division of Hepatobiliary, Department of Internal Medicine, Faculty of Medicine, University of Indonesia, Jakarta, Indonesia; 6 Division of Hematology and Medical Oncology, Department of Internal Medicine, Faculty of Medicine, University of Indonesia, Jakarta, Indonesia

**Keywords:** Hepatitis, immune checkpoint inhibitor, immunotherapy, review

## Abstract

Immunotherapy using immune checkpoint inhibitor (ICI) has been increasingly used in the oncology treatment field. Although ICIs could help suppress cancer and improve survival rates, it could also lead to certain adverse events, including immune-mediated liver injury caused by ICIs (ILICI). The manifestation of ILICI ranged greatly from asymptomatic disease to liver failure and even death. In this review article, we will discuss the pathogenesis, manifestation, and clinical approach of ILICI.

## INTRODUCTION

Immunotherapy has revolutionized the landscape of cancer management, especially in terms of precision medicine. In this context, “precision” also includes individualized risk assessment and management of immune-related toxicities—an important determinant of whether patients can safely continue effective immune checkpoint inhibitor (ICI) therapy. Increasing numbers of new ICIs have been discovered, along with their approved indications for various types of cancer.^1^

Immune checkpoint inhibitors modulate immune checkpoint pathways, which can lead to tumor regression and durable disease control in selected patients. There are three ICI classes: cytotoxic T lymphocyte-associated protein-4 (CTLA-4) inhibitors, programmed cell death protein 1 (PD-1) inhibitors, and programmed death-ligand 1 (PD-L1) inhibitors. Immune checkpoint inhibitors can be administered either alone or in combination with other cancer therapies such as surgery, conventional chemotherapy, radiotherapy, and other modalities.^2^

The widespread use of ICIs has been associated with improved prognosis and quality of life of specific groups of cancer patients, including melanoma, non-small cell lung cancer (NSCLC), breast cancer, renal cell carcinoma, Hodgkin lymphoma, head and neck cancer, and urothelial carcinoma.^3^ However, as use expands, the incidence of ICI-related toxicity is expected to increase. Although ICI toxicities can be acute or long-term, they are typically associated with immune reactions and present acutely. These toxicities may lead to significant morbidity, impaired quality of life, and abrupt discontinuation of ICI therapy. The toxicity of ICIs varies depending on the ICI class and may affect various organs. In a previous paper, we discussed ICI-induced colitis.^4^ This narrative review discusses ICI hepatitis, a common ICI immune-related adverse event (irAE) affecting the liver.

## METHODS

This narrative review synthesizes current evidence on immune-mediated liver injury caused by ICIs (ILICI), with emphasis on recent clinical guidelines and management strategies. A comprehensive literature search was conducted using PubMed, Embase, and Web of Science databases from inception through October 2024. Search terms included combinations of “immune checkpoint inhibitor,” “immunotherapy,” “hepatotoxicity,” “hepatitis,” “liver injury,” “PD-1,” “PD-L1,” “CTLA-4,” and related terms. Randomized controlled trials, observational studies, case series, clinical practice guidelines, and expert consensus statements were included. Priority was given to current guidelines from major societies. This review provides an updated synthesis of ILICI epidemiology, pathogenesis, clinical presentation, diagnosis, and management, based on the included literature. An emphasis is placed on the conceptual framework of ILICI as a distinct form of indirect drug-induced liver injury. Hence, a detailed comparative analysis of management recommendations across guidelines is provided, with particular attention given to special populations, including patients with hepatocellular carcinoma, chronic viral hepatitis, autoimmune liver disease, and liver transplant recipients—groups that are often underrepresented in clinical trials but frequently encountered in practice. This emphasis supports a practical precision-oncology approach to risk stratification, monitoring, and management, discussed herein.

## DEFINITIONS

Several different terms have been used to describe ICI hepatitis, including ILICI, immune-mediated hepatitis (IMH) induced by ICI, checkpoint inhibitor-induced liver injury (CHILI), and immune checkpoint inhibitor-related hepatotoxicity (ICH).^5^–^7^

While ILICI can be seen as part of drug-induced liver injury (DILI), many experts consider ILICI a distinct type of DILI. Classically, DILI is divided into two types: intrinsic (direct) and idiosyncratic. Intrinsic DILI is usually predictable, dose-dependent, and has rapid onset after drug initiation. Idiosyncratic DILI, on the other hand, is dose-independent, unpredictable, and may have delayed onset.^8^ The DILI Initiative of the International Consortium for Innovation and Quality in Pharmaceutical Development proposed that ILICI represents a third DILI category, distinct from the traditional intrinsic (direct) and idiosyncratic types. This proposal was based on its distinct mechanism, clinical presentation, and response to immunosuppression as well as the indirect, immune-mediated effects of ICIs on the liver.^6^ In 2023, the American Association for the Study of Liver Diseases (AASLD) also proposed a third type of DILI called “indirect hepatotoxicity.” Accordingly, ILICI has been classified within this third DILI type. In contrast to intrinsic and idiosyncratic DILI, indirect hepatotoxicity is partially predictable, dose-independent, and may have a latency for months. Additionally, it arises when the biological action of the drug affects the host immune system, leading to a secondary form of immune-mediated liver injury.^9^

## EPIDEMIOLOGY

During ICI therapy, excessive T cell activation and reduced regulatory T cell function can trigger immune-related adverse events across multiple organs, including ILICI. The incidence of ILICI has increased in recent years, making it the third most common ICI-related adverse effect (up to 30%) after dermatologic and gastrointestinal toxicity.^7^,^10^–^12^ In a retrospective study, Hountondji et al. observed three distinct clinical patterns of ILICI, namely hepatocellular (38.5%), cholestatic (36.8%), and mixed (24.8%); no severe acute cases were seen.^5^ Other studies have also shown that the hepatocellular pattern was the most commonly observed pattern in patients with ILICI.^7^

Cases of ILICI are characterized by a significant increase in transaminases, followed by a gradual or rapid decrease.^13^ The onset of transaminase elevation usually occurs 4 to 12 weeks following the initiation of ICI treatment, or after receiving one to three doses of ICI.^14^–^18^ Importantly, ILICI can present with a delayed onset, occurring several months after treatment initiation or even after treatment cessation.^9^–^22^ Cases have been reported as early as 2–3 weeks and as late as 21 months after initiation.^16^,^19^,^20^ Delayed-onset cases have been documented, occurring 7–9 weeks or even up to 24 months after the last dose of ICI, emphasizing the need for prolonged clinical vigilance and monitoring.^19^–^22^ Clinicians should maintain a high index of suspicion for ILICI in patients with prior ICI exposure, even months after treatment discontinuation, and monitoring for immune-related adverse events, including liver function, should be individualized and may extend up to 12 months post-treatment cessation.^21^,^22^

Liver injury with a mixed pattern is usually seen at the beginning, while the hepatocellular injury pattern is seen at its peak. Fever may also be a clinical manifestation. In rare cases, acute liver failure is the first presentation.^16^

Distinct patterns of liver injury have been observed with CTLA-4 versus PD-1/PD-L1 inhibitors: ILICI associated with anti-CTLA-4 is often more severe than with anti-PD-1 and anti-PD-L1. Most ILICI cases are mild, but if they are not treated properly, there is a risk of acute liver failure and even death. In addition, inappropriate ILICI management can lead to the failure of cancer therapy. Therefore, ILICI has become an increasing concern.^10^

It is important to note that published studies use heterogeneous definitions of ILICI, ranging from any degree of liver enzyme elevation to clinically significant immune-mediated hepatitis requiring immunosuppression (e.g. Common Terminology Criteria for Adverse Events [CTCAE] grade ≥3). This variability contributes substantially to the wide range of incidence figures reported in the literature. The incidence of ILICI varies according to the type of ICI.^11^,^23^ Hepatotoxicity with PD-1 inhibitors was found to be between 1% and 3%, while the incidence of various grades of autoimmune hepatotoxicity with CTLA-4 inhibitors has been reported between 3% and 9%.^14^ Furthermore, combination therapy is associated with a much higher incidence of hepatotoxicity, with incidence rates ranging from 13% to 30% for all grades and 6% to 19% for grade 3 or higher.^6^,^13^,^14^,^24^,^25^ The incidence of the different agents of ICI have been listed in Table 1. These wide incidence ranges largely reflect the heterogeneity of case definitions, differences in monitoring frequency, and variable attribution methods used across clinical trials and observational studies.

**Table 1:** List of ICI agents and incidence of ILICI.

Drug^ref^	Incidence of ILICI*
PD-1 inhibitor	
Nivolumab^22^,^26^–^31^	2%–11%
Pembrolizumab^32^–^42^	0.7%–26.8%

PD-L1 inhibitor	
Atezolizumab^43^–^54^	4%–35.9%
Durvalumab^55^	2.1%
Avelumab^56^–^58^	3.3%–17.7%

CTLA-4 inhibitor	
Ipilimumab^59^–^62^	3.8%–59.2%
Tremelimumab^63^	6%

* Ranges reflect reported incidence across studies; definitions and regimens vary.

CTLA-4, cytotoxic T lymphocyte-associated protein-4; ICI, immune checkpoint inhibitor; ILICI, immune-mediated liver injury caused by immune checkpoint inhibitor; PD-1, programmed cell death protein 1; PD-L1, programmed death ligand 1.

## RISK FACTORS

Numerous risk factors—including the type and dosage of ICI, whether it is used alone or in conjunction with other ICIs or small molecule inhibitors, genetic predisposition, and concomitant medications (e.g. acetaminophen and statins)—could influence the occurrence of ILICI.^13^,^21^ The different risk factors for ILICI are summarized in Box 1.

Box 1: **Risk Factors for ILICI**

- Female
- Younger age
- Anti-CTLA-4 > anti-PD-1 > anti-PD-L1
- Use of ≥2 ICI agents
- Higher dose*
- Pre-existing liver disease (hepatitis B and C)
- Liver malignancy
- Autoimmune disease

*Only applies to anti-CTLA-4 agents; anti-PD-1 and anti-PD-L1 are not dose-dependent.

Several studies have reported a higher incidence of ILICI in patients treated with two or more ICIs compared to a single agent. The incidence of increased aminotransferase (AST)/alanine transaminase (ALT) in those receiving combination therapy ranged from 4.0% to 22.3% compared to 1.7%–12.0% in the monotherapy group. The incidence of grade 3 or 4 hepatic irAEs was also greater in the combination group (6.1%–14.9% versus 0%–1%).^64^–^67^ A meta-analysis of 17 clinical trials found that those receiving anti-CTLA-4 agents had higher odds for hepatotoxicity (anti-CTLA-4 versus control: odds ratio [OR] 4.67, 95% CI 3.42–6.39; anti-PD-1 versus control: OR 1.58, 95% CI 0.66–3.78; *P* value for anti-CTLA-4 versus anti-PD-1: <0.00001), and elevation in AST/ALT when compared to anti-PD-1 agents (AST elevation in anti-CTLA-4 versus control compared to anti-PD-1 versus control: OR 3.36 versus 2.10, *P* value for anti-CTLA-4 versus anti-PD-1: <0.00001; ALT elevation in anti-CTLA-4 versus control compared to anti-PD-1 versus control: OR 4.45 versus 2.13, *P* value for anti-CTLA-4 versus anti-PD-1: <0.00001).^68^ Compared to anti-PD-1 agents, anti-PD-L1 also had a lower incidence for elevations in AST and ALT (AST: 6.84% versus 3.72%, *P*<0.001; ALT: 6.01% versus 3.60%, *P*<0.001).^69^ A higher dosage of anti-CTLA-4 has been linked to a higher incidence of hepatitis (ipilimumab 3 mg/kg: 3%–5%; ipilimumab 10 mg/kg: 15%–16%).^70^ On the other hand, hepatic irAEs due to anti-PD-1/PD-L1 did not seem to be dose-dependent.^71^

The risk of ILICI also appeared to vary by cancer type, with reported incidences expressed as the percentage of patients experiencing aminotransferase elevation. Patients with hepatocellular carcinoma have been reported to have a higher incidence of ALT elevation (8%) compared with other cancers, such as lung cancer (0%) and melanoma (0%–4%).^65^,^72^–^77^ A meta-analysis of 117 studies also confirmed that patients with liver cancer had a higher incidence of hepatotoxicity compared to other solid tumors (ALT increase: 13.2% [95% CI 8.54%–20.4%] versus 4.92% [95% CI 4.21%–5.76%]; AST increase: 14.2% [95% CI 9.93%–20.4%] versus 5.38% [95% CI 4.52%–6.39%]). In addition, the incidence of elevated aminotransferase levels that were of grade 3 or above was also higher in the liver cancer group compared to other solid tumors (ALT increase: 4.57% [95% CI 3.38%–6.17%] versus 1.26% [95% CI 1.02%–1.56%], *P*<0.001; AST increase: 6.74% [95% CI 4.09%–11.11%] versus 1.19% [95% CI 0.95%–1.48%], *P*<0.001).^69^ Overall, hepatocellular carcinoma patients may have a 2–3-fold higher risk of ILICI compared to other cancer types, and underlying chronic liver disease and cirrhosis may contribute to increased susceptibility. Therefore, baseline liver function assessment (Child–Pugh score) should be performed before ICI initiation, and more frequent monitoring of liver function may be warranted in hepatocellular carcinoma patients on ICIs. It is also important to distinguish ILICI from tumor progression, portal vein thrombosis, or decompensation of underlying liver disease.

One cohort study observed that females were more likely to experience ILICI than were males (OR 2.54, 95% CI 1.09–6.06, *P*=0.03).^78^ This was also reported by another study involving 1096 participants (*P*=0.038).^79^ A meta-analysis of 13 studies indicated that younger age was significantly associated with higher incidence of ILICI (weighted mean difference [WMD]: −5.200, 95% CI −7.481 to −2.919) and grade 3 or above ILICI (WMD: −5.193, 95% CI −9.669 to −0.718).^80^

The presence of pre-existing liver diseases, such as hepatitis B virus (HBV) or hepatitis C virus (HCV) infection, is thought to be a risk factor for hepatotoxicity when receiving ICIs. Animal studies have shown that ICI exposure in HBV- or HCV-infected animals was associated with elevated aminotransferase levels.^81^,^82^ The available data on humans are limited, since most studies excluded patients with pre-existing liver conditions. A case series of nine individuals with HBV or HCV infection who received ICIs experienced elevations in aminotransferase levels.^83^ Cirrhotic patients with HCV infection treated with tremelimumab also had a higher incidence of ALT elevation than did those without HCV (25% versus 3%).^84^ Although these few human studies suggest an association between hepatitis infection and a higher risk for hepatotoxicity, elevations in transaminase levels may also be due to the underlying hepatitis infection rather than ICI exposure. Thus, patients with chronic HBV should be considered for antiviral prophylaxis before ICI initiation to prevent reactivation. Reactivation of HBV can occur during or after ICI therapy; therefore HBV DNA should be monitored regularly.^85^ Chronic HCV infection does not appear to increase ILICI risk significantly; successful HCV treatment prior to ICI is preferred when feasible. Appropriate serological and virological testing should be carried out to distinguish ILICI from viral hepatitis reactivation.^86^

Very few studies have assessed the safety of ICIs in individuals with liver transplants. One of the concerns regarding ICI usage in this population is the risk of allograft rejection. In one study, as many as 7 out of 19 patients (39%) experienced allograft rejection, with the highest rates of rejection seen in those receiving combination therapy (50%), followed by nivolumab (33%), pembrolizumab (25%), and ipilimumab (12.5%) monotherapy.^87^ On the other hand, several other cases have reported tolerability of ICIs in solid-organ transplant recipients.^88^–^90^ Such mixed results make it difficult to conclude the safety of ICIs in liver transplant recipients. However, given the high rate of allograft rejection, ICIs should be used cautiously, with careful risk-benefit assessment.

Patients with autoimmune diseases are another unique population that requires special attention with ICI usage. Studies have found that autoimmune patients have a notably higher occurrence of irAEs (29%–45%) and disease exacerbation (29%–47%) when on ICIs.^91^–^96^ Despite the higher irAE rates, these events did not have a significant impact on overall survival.^92^ Furthermore, most of the cases were easily resolved without discontinuing ICIs.^94^ Therefore, pre-existing autoimmune disease is not an absolute contraindication to ICI therapy. More frequent monitoring for both disease flares and immune-related adverse events are recommended. Multidisciplinary management is also recommended.^95^

## PATHOGENESIS

### Indirect Hepatotoxicity Mechanism of DILI

As previously stated, a third subtype mechanism of DILI—the indirect hepatotoxicity subtype—has been proposed.^8^–^9^ This third mechanism was mainly attributed to the effects of a drug towards the host’s immune response.^9^ This type of DILI is mainly described in ILICI patients and in those who experience HBV reactivation after administration of immunosuppressants.^97^ This third type can be distinguished from direct hepatotoxicity and idiosyncratic hepatotoxicity based on its distinct mechanisms.^8^,^9^

### Immune-mediated Liver Injury Caused by ICIs

In ILICI, T cell activation and loss of tolerance against the patient’s own cells lead to liver injury.^10^ The mechanism by which ICIs elicit ILICI varies by class. For example, anti-CTLA-4 agents affect T cells primarily at the priming stage, whereas anti-PD-1/PD-L1 act mainly at the effector stage.^10^,^98^ During the priming stage, CTLA-4 on T cells competitively binds B7-1 and B7-2 on antigen-presenting cells, thereby inhibiting CD28-mediated T cell activation.^10^ Anti-CTLA-4 agents bind to CTLA-4 on T cells and block this inhibitory signal, promoting T cell activation.^10^,^99^

The binding of PD-1 on T cells to PD-L1 on tumor cells promotes evasion by inhibiting T cell activation.^10^ Overactivation of T cells leads to clonal expansion of Th1 and Th17 CD4+ T cells, which produce proinflammatory cytokines, such as IL-2, IFN-γ, and TNF-α.^10^,^98^ These cytokines will then activate the innate immune system, as well as CD8+ cytotoxic T lymphocytes, leading to increased production of intracellular granzyme B and perforin.^99^ Overactivation of CD8+ T cells also contributes to overcoming immune tolerance and hepatocyte injury.^98^ In addition, regulatory T cells (Tregs) are also suppressed, resulting in reduced production of anti-inflammatory cytokines (IL-10, IL-35, and TGF-β) and a proinflammatory environment.^10^,^98^

### Comparison of ILICI versus Other Type of DILI

In general, direct DILI occurs due to an imbalance between toxin production and the detoxification capacity of hepatocytes, leading to increased oxidative stress and mitochondrial dysfunction.^98^ On the other hand, the pathogenic mechanisms of idiosyncratic drug-induced liver injury (iDILI) and ILICI are more similar to one another, in that both involve overactivation of the innate and adaptive immune systems.^99^,^100^ However, iDILI occurs due to the production of neoantigens following drug metabolism; inflammation is only triggered once hepatocyte damage occurs.^99^,^100^ Meanwhile, ILICI occurs due to ICI exposure, which inhibits the ability of CTLA-4, PD-1, and PD-L1 to suppress T cell activation.^10^,^101^

## CLINICAL MANIFESTATION

The manifestation of ILICI ranges from asymptomatic to acute liver failure. Most ILICI cases are asymptomatic and diagnosed incidentally when monitoring for liver function tests after ICI therapy. Those with more severe disease may present with right upper quadrant abdominal pain, fever, fatigue, rash, jaundice, dark urine, and easy bruising.^102^–^106^ Although this is rare, patients with ILICI may also manifest with acute liver failure during the initial stages.^103^ Some common grading systems used to classify ILICI severity are the CTCAE and the Drug-induced Liver Injury Network (DILIN) grading systems (Table 2).^107^,^108^

**Table 2:** Grading of ILICI Severity According to Two Common Grading Systems.^107^,^108^

Grade^*^	CTCAE	DILIN
**Grade 1**	ALT >ULN to ≤3× ULN AST >ULN to ≤3× ULN Total serum bilirubin ULN to ≤1.5× ULN ALP ULN to ≤2.5× ULN	Elevation in ALT and/or ALP levels Total serum bilirubin <2.5 mg/dL INR <1.5 Present with or without symptoms (nausea, vomiting, asthenia, fatigue, RUQ pain, jaundice, rash, pruritus, weight loss)
**Grade 2**	ALT >3–5× ULN AST >3–5× ULN Total serum bilirubin >1.5–3× ULN ALP >2.5–5× ULN	Elevation in ALT and/or ALP levels Total serum bilirubin ≥2.5 mg/dL or INR ≥1.5 Symptoms may become aggravated
**Grade 3**	ALT >5–20× ULN AST >5–20× ULN Total serum bilirubin >3–10× ULN ALP >5–20× ULN	Elevation in ALT, ALP, and total serum bilirubin ≥2.5 mg/dL and/or INR ≥1.5 Symptoms are further aggravated Indication for hospitalization No evidence of hepatic encephalopathy
**Grade 4**	ALT >20× ULN AST >20× ULN Total serum bilirubin >10× ULN ALP >20× ULN	Elevation in ALT, ALP, and total serum bilirubin ≥2.5 mg/dL Signs of hepatic failure (INR ≥1.5, ascites, hepatic encephalopathy) and/or DILI-related dysfunction of another organ
**Grade 5**	Death/mortality due to ILICI	Death/mortality due to ILICI OR Requires liver transplantation for survival

Note: CTCAE and DILIN grades are not directly interchangeable.

* Severity grade is determined by the highest grade for which at least one criterion is met.

ALP, alkaline phosphatase; ALT, alanine transaminase; AST, aspartate aminotransferase; CTCAE, Common Terminology Criteria for Adverse Events; DILI, drug-induced liver injury; DILIN, Drug-induced Liver Injury Network; dL, deciliter; INR, international normalized ratio; mg, milligram; RUQ, right upper quadrant; ULN, upper limit of normal.

## DIAGNOSTIC APPROACH

Clinically, ILICI is often asymptomatic, but it may occasionally present with abdominal pain in the right upper quadrant, accompanied by fever, rash, fatigue, dark urine, and jaundice. Clinicians should review the patient’s medication history, including the ICI agent used and duration of therapy. Typically, ILICI occurs within 4–12 weeks of starting ICIs or after approximately three ICI infusions. Importantly ILICI is a diagnosis of exclusion; therefore, alternative causes should be ruled out, including hepatotoxicity from other medications (e.g. acetaminophen), viral hepatitis, other infections, tumor-related liver involvement, biliary disease, autoimmune hepatitis, myositis, and rhabdomyolysis. The recommended workup is provided in Box 2.^5^,^7^,^109^

Box 2: **Diagnostic Workup for ILICI^7^,^109^**

- Complete blood count
- ALT, AST, total serum bilirubin, ALP
- INR
- Viral hepatitis panel (anti-HAV IgM, HBsAg, anti-HBc IgM and IgG ± HBV DNA, anti-HCV ± HCV RNA, anti-HEV IgM, EBV IgM and IgG, CMV IgM and IgG ± CMV DNA)
- Autoimmune panel (ANA, ASMA, anti-LKM1, serum IgG)
- Serum CK
- Iron studies (ferritin, transferrin saturation)
- Abdominal imaging (CT scan, MRI, or USG with Doppler)

**Abbreviations:** ALP, alkaline phosphatase; ALT, alanine transaminase; ANA, anti-nuclear antibodies; Anti-HBc, anti-Hepatitis B core antibody; Anti-LKM1, anti-liver-kidney microsomal 1 antibody; ASMA, anti-smooth muscle antibody; AST, aspartate aminotransferase; CK, creatine kinase; CMV, cytomegalovirus; CT, computed tomography; DNA, deoxyribonucleic acid; EBV, Epstein-Barr virus; HAV, hepatitis A virus; HBV, hepatitis B virus; HCV, hepatitis C virus; HEV, hepatitis E virus; IgG, immunoglobulin G; IgM, immunoglobulin M; INR, international normalized ratio; MRI, magnetic resonance imaging; RNA, ribonucleic acid; USG, ultrasonography

One of the most common presentations of ILICI is abnormal liver function tests. The *R* value, defined as the ratio of ALT to ALP after normalization to their upper limit of normal (ULN), [*R* = (*ALT/ULN*)/(*ALP/ULN*)], can be used to determine the pattern of liver injury. There are three patterns: cholestatic (*R*≤2), hepatocellular (*R*≥5), and mixed (2<*R*<5).^5^ The hepatocellular pattern (60%) is the most common presentation in ILICI. However, cholestatic (30%) or mixed (10%) patterns are more common in patients receiving anti-PD-1/PD-L1 compared with anti-CTLA-4. Elevated total serum bilirubin may also be observed in ILICI and may indicate greater severity.^7^,^12^ Due to limited reports of cholestatic-type ILICI, its pattern of occurrence and risk factors are not well defined. This pattern is typically characterized by predominant elevation of ALP and gamma-glutamyl transferase, a more severe disease course, less responsiveness to corticosteroid therapy, and may be associated with a poorer prognosis compared to hepatocellular patterns.^110^–^114^

Abdominal imaging may be useful in excluding other potential diagnoses such as metabolic dysfunction-associated steatohepatitis, portal vein/hepatic vein thrombosis, ischemic hepatitis, and hepatic tumors.^7^ Magnetic resonance cholangiopancreatography may be recommended when evaluating cholestatic-pattern liver injury, as it can help identify biliary abnormalities, including possible biliary strictures.^6^,^114^

Similarly, liver biopsy might also be considered to exclude differential diagnoses and evaluate the disease severity.^9^ However, this examination is invasive and expensive; hence, clinicians should consider the risks and benefits.^9^,^115^ Liver biopsy is not routinely required for ILICI diagnosis but should be considered in specific clinical circumstances. Liver biopsy may be recommended when: (1) patients fail to improve after empirical therapy; (2) bilirubin levels are elevated without radiographic evidence of biliary obstruction; (3) clinical features are atypical, or the clinical course is unusual; (4) CTCAE grade 2 or 3 is present; (5) exclusion of other etiologies is needed, including malignant biliary obstruction, diffuse hepatic metastases, drug-induced liver injury from concurrent medications, or opportunistic viral infections; or (6) patients with cholestatic patterns require differentiation from primary biliary cholangitis or malignant biliary obstruction.^115^–^120^ The most common histological patterns include panlobular hepatitis (hepatocellular pattern) with lobular inflammation and hepatocyte injury, or portal-based inflammation with bile duct injury (cholangiopathy pattern),^115^,^117^,^118^ though no pathognomonic findings exist exclusively for ILICI.^9^,^115^

**Figure 1:**
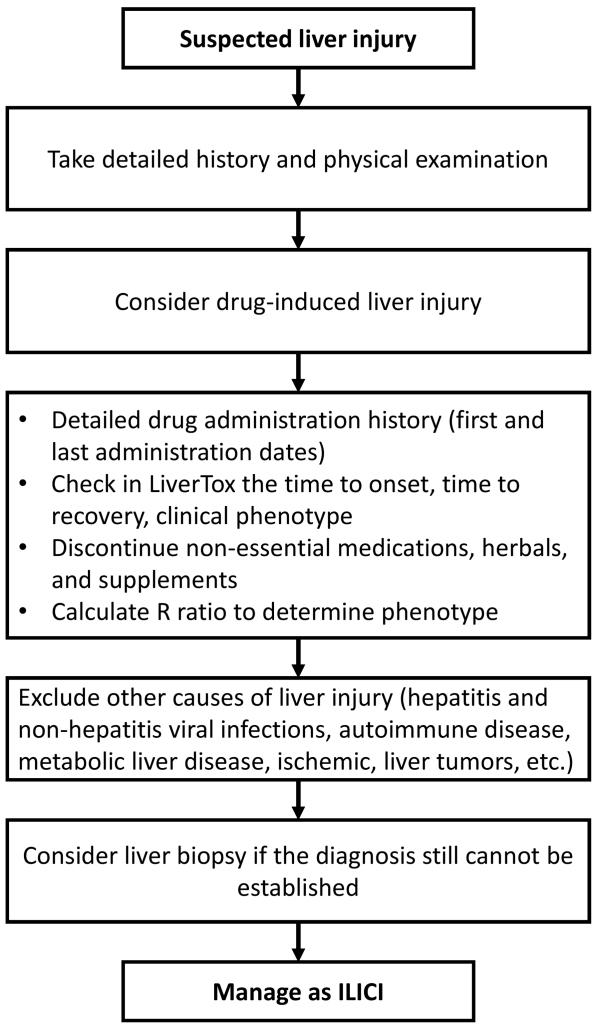
Diagnostic Flowchart for Immune-mediated Liver Injury Caused by Immune Checkpoint Inhibitors (ILICI).^9^,^122^

Histologic findings in ILICI are characterized by periportal and lobular inflammation, hepatocyte dropout, and centrilobular necrosis. Although the infiltrates can be mixed, they are usually dominated by T lymphocytes and histiocytes with few or no plasma cells.^7^,^121^ Other sources have described the pathological features of ILICI as panlobular hepatitis (lobular inflammation: lymphocytes and macrophages), cholestatic pattern (portal-based inflammation with bile duct injury: lymphocytes, plasma cells, neutrophils, and eosinophils), and mixed pattern.^121^ Figure 1 summarizes the diagnostic approach for ILICI.^9^,^122^

## MANAGEMENT APPROACH

Several societies have published guidance regarding the diagnosis and management of ILICI. This includes the AASLD,^9^ American Gastroenterology Association (AGA),^116^ American Society of Clinical Oncology (ASCO),^119^ European Association for the Study of the Liver (EASL),^8^ European Society for Medical Oncology (ESMO),^123^ Multinational Association of Supportive Care in Cancer (MASCC),^124^ National Comprehensive Cancer Network (NCCN),^125^ and Society for Immunotherapy of Cancer (SITC).^126^ A comparison of these guidelines is summarized in Supplementary Table 1

### Grade 1 Hepatotoxicity

For grade 1 hepatotoxicity, the ILICI guidelines recommend continuing ICIs, especially if the condition is asymptomatic.^8^,^9^,^13^,^116^,^119^,^123^ When symptomatic, clinicians may give symptomatic treatment while monitoring the patient’s condition closely, including periodic liver testing.^8^,^9^,^13^,^116^,^119^,^125^,^126^ Clinicians are advised to also perform other tests to eliminate other possible causes of hepatitis. These include testing for viral hepatitis infection, human immunodeficiency virus, autoimmune etiologies (e.g. antinuclear antibody, anti-smooth muscle antibody, antineutrophil cytoplasmic antibody, anti-mitochondrial antibody), iron studies (e.g. iron, ferritin, total-iron binding capacity), and radiologic evaluation for biliary obstruction.^8^,^116^,^119^,^123^–^126^ It is also important to reassess the patient’s history of alcohol consumption and withhold other potentially hepatotoxic medications.^8^,^116^,^119^,^123^–^126^

### Grade 2 Hepatotoxicity

All eight guidelines agree to temporarily withhold ICIs in grade 2 hepatotoxicity.^8^,^9^,^116^,^119^,^123^–^126^ All except for EASL agree to start 0.5–1.0 mg/kg/day oral prednisone.^9^,^116^,^119^,^123^–^126^ Both ASCO^119^ and MASCC^124^ suggest adding immunosuppressive therapy, such as mycophenolate mofetil, if the patient does not respond to steroid therapy. Resumption of ICI therapy may be considered once the corticosteroids have been tapered to ≤10 mg/day prednisone (or equivalent) over 2–4 weeks and hepatotoxicity has improved to grade ≤1.^8^,^116^,^119^,^123^–^126^ Several of the guidelines also recommend monitoring liver parameters, international normalized ratio (INR), and albumin every 3–7 days.^8^,^116^,^123^,^124^,^126^ The AGA,^116^ ASCO,^119^ MASCC,^124^ and SITC^126^ guidelines also suggest considering liver biopsy in grade 2 hepatotoxicity to confirm the underlying pathology. The ESMO^123^ and MASCC^124^ guidelines only recommend screening for other possible causes of hepatitis starting from grade 2 hepatotoxicity or higher. Meanwhile, other guidelines from AASLD,^9^ AGA,^116^ ASCO,^119^ EASL,^8^ NCCN,^125^ and SITC^126^ recommend this examination starting from grade 1 hepatotoxicity.

### Grade 3 or 4 Hepatotoxicity

The AASLD, AGA, ASCO, EASL, and SITC all recommend permanently stopping ICIs in grade 3 or higher hepatotoxicity.^9^,^116^,^119^,^126^ However, MASCC did not specify whether ICIs should be temporarily or permanently discontinued in grade ≥3 hepatotoxicity.^124^ Meanwhile, ESMO and NCCN both suggest withholding ICIs temporarily in grade 3 hepatotoxicity and permanently discontinuing ICIs in grade 4 hepatotoxicity. If the patient’s condition has improved to grade 1 hepatotoxicity, ICIs can be resumed.^123^,^125^ All guidelines agree to administer intravenous steroids, such as methylprednisolone, in grade 3–4 hepatotoxicity, but with varying doses. The most commonly recommended dose was 1–2 mg/kg/day of intravenous methylprednisolone or its equivalent.^8^,^116^,^119^,^125^ Other guidelines such as NCCN suggest 1.0 mg/kg/day, AASLD suggests 1–1.5 mg/kg/day, and MASCC recommends 0.5–2 mg/kg/day.^9^,^124^,^125^ Meanwhile, ESMO recommended giving 1 mg/kg/day if the AST/ALT levels were <400 U/L and the patient had normal bilirubin, INR, and albumin. Otherwise, 2 mg/kg/day should be administered.^123^ If the patient is refractory to steroids, immunosuppressive regimens, such as mycophenolate mofetil, tacrolimus, or azathioprine, can be given.^8^,^9^,^116^,^119^,^123^–^126^ Antithymocyte globulin may also be considered in patients with fulminant hepatitis.^116^,^124^,^126^ Patients with grade ≥3 hepatotoxicity should also be hospitalized, possibly referred to a hepatologist, and undergo routine liver tests every 1–3 days.^8^,^9^,^116^,^119^,^123^–^126^ Whenever possible, a liver biopsy should also be considered at this stage.^8^,^116^,^119^,^123^–^126^

Based on the synthesis of current guidelines and clinical evidence, we propose the practical approach to ILICI management shown in Table 3.

**Table 3:** Proposed Practical Approach for Management of Immune-mediated Liver Injury Caused by Immune Checkpoint Inhibitors (ILICI).

ILICI Grade	Management
**Grade 1**	Continue immune checkpoint inhibitors (ICI) Close monitoring Investigate other possible causes of hepatitis and withhold hepatotoxicity drugs *If asymptomatic*, no specific treatment is required *If symptomatic*, continue ICI unless symptoms are concerning or worsening. Give symptomatic treatment and frequent monitoring. Manage as grade 2 if clinical condition worsens
**Grade 2**	Temporarily withhold ICI Investigate other possible causes of hepatitis and withhold hepatotoxicity drugs Initiate prednisone 0.5–1.0 mg/kg/day Monitor liver function every 3 days *If no improvement*, consider liver biopsy and start adding immunosuppressant *If there is improvement*, begin gradual steroid taper over 4–6 weeks Consider ICI rechallenge after resolution, with close monitoring
**Grade 3**	Permanently discontinue ICI Investigate other possible causes of hepatitis and withhold hepatotoxicity drugs Hospitalization and close monitoring Start IV methylprednisolone 1–2 mg/kg/day Monitor liver function every 1–2 days Consider liver biopsy if not previously performed *If no improvement*, add immunosuppressant: Preferred first line, mycophenolate mofetil; preferred second line, azathioprine, tacrolimus; preferred as rescue therapy, anti-thymocyte globulin Once improved to grade ≤1, begin gradual steroid taper over 4–6 weeks
**Grade 4**	Same management as grade 3 IV methylprednisolone can be considered, starting at 2 mg/kg/day Monitor liver function daily

## CONCLUSION

As ICI use continues to expand, ILICI may become an increasing clinical issue. Therefore, clinicians should consider ILICI in patients who develop abnormal liver function tests after initiating ICIs. Further studies are needed to refine diagnostic and therapeutic approaches for ILICI.

## Supplementary Information



## Abbreviations

AASLD: American Association for the Study of Liver Disease
ALT: alanine transaminase
AST: aspartate aminotransferase
CHILI: checkpoint inhibitor-induced liver injury
CTCAE: Common Terminology Criteria for Adverse Events
CTLA-4: cytotoxic T lymphocyte-associated protein-4
DILI: drug-induced liver injury
HBV: hepatitis B virus
HCV: hepatitis C virus
ICH: immune checkpoint inhibitor-related hepatotoxicity
ICI(s): immune checkpoint inhibitor(s)
ILICI: immune-mediated liver injury caused by ICIs
IMH: immune-mediated hepatitis
PD-1: programmed cell death protein 1
PD-L1: programmed death ligand 1.

## References

[1] Alturki NA (2023). Review of the immune checkpoint inhibitors in the context of cancer treatment. J Clin Med.

[2] Shiravand Y, Khodadadi F, Kashani SMA (2022). Immune checkpoint inhibitors in cancer therapy. Curr Oncol.

[3] Perdyan A, Sobocki BK, Balihodzic A, Dąbrowska A, Kacperczyk J, Rutkowski J (2023). The effectiveness of cancer immune checkpoint inhibitor retreatment and rechallenge-a systematic review. Cancers (Basel).

[4] Adiwinata R, Tandarto K, Tanadi C (2024). Immune checkpoint inhibitor colitis, a rising issue in targeted cancer therapy era: a literature review. Rom J Intern Med.

[5] Hountondji L, Ferreira De Matos C, Lebossé F (2023). Clinical pattern of checkpoint inhibitor-induced liver injury in a multicentre cohort. JHEP Rep.

[6] Regev A, Avigan MI, Kiazand A (2020). Best practices for detection, assessment and management of suspected immune-mediated liver injury caused by immune checkpoint inhibitors during drug development. J Autoimmun.

[7] Remash D, Prince DS, McKenzie C, Strasser SI, Kao S, Liu K (2021). Immune checkpoint inhibitor-related hepatotoxicity: a review. World J Gastroenterol.

[8] European Association for the Study of the Liver (2019). EASL clinical practice guidelines: drug-induced liver injury. J Hepatol.

[9] Fontana RJ, Liou I, Reuben A (2023). AASLD practice guidance on drug, herbal, and dietary supplement-induced liver injury. Hepatology.

[10] Liu Z, Zhu Y, Xie H, Zou Z (2022). Immune-mediated hepatitis induced by immune checkpoint inhibitors: current updates and future perspectives. Front Pharmacol.

[11] Velarde-Ruiz Velasco JA, Tapia Calderón DK, Cerpa-Cruz S (2024). Immune-mediated hepatitis: basic concepts and treatment. Rev Gastroenterol Mex (Engl Ed).

[12] Zheng C, Huang S, Lin M (2023). Hepatotoxicity of immune checkpoint inhibitors: what is currently known. Hepatol Commun.

[13] Hercun J, Vincent C, Bilodeau M, Lapierre P (2022). Immune-mediated hepatitis during immune checkpoint inhibitor cancer immunotherapy: lessons from autoimmune hepatitis and liver immunology. Front Immunol.

[14] Hernandez N, Bessone F (2022). Hepatotoxicity induced by biological agents: clinical features and current controversies. J Clin Transl Hepatol.

[15] Kuo L, Kuwelker S, Tsai E (2023). Management of autoimmune and viral hepatitis in immunotherapy: a narrative review. Ann Palliat Med.

[16] Peeraphatdit TB, Wang J, Odenwald MA, Hu S, Hart J, Charlton MR (2020). Hepatotoxicity from immune checkpoint inhibitors: a systematic review and management recommendation. Hepatology.

[17] Pham C, Ennin E, Enriquez K (2024). Immune-mediated liver injury from checkpoint inhibitors requiring supratherapeutic corticosteroid and mycophenolate treatment. AIM Clin Cases.

[18] Puzanov I, Diab A, Abdallah K (2017). Managing toxicities associated with immune checkpoint inhibitors: consensus recommendations from the Society for Immunotherapy of Cancer (SITC) Toxicity Management Working Group. J Immunother Cancer.

[19] Barry C, Huang J, Ubanatu CB, Bozym J (2025). Delayed-onset immune-mediated hepatitis following pembrolizumab discontinuation: a case report. Cureus.

[20] Chopra M, Zhang HC (2023). S3887 Recognition of delayed-onset immune checkpoint inhibitor-mediated hepatotoxicity treated with budesonide: a case study factoring in pharmacokinetics of immunotherapies. Am J Gastroenterol.

[21] Echeverri-Hoyos J, Tuta-Quintero E, Echeverri Franco JA, Bonilla N, Rojas-Londoño C (2025). Late-onset immune-mediated hepatotoxicity induced by the immune checkpoint inhibitor pembrolizumab in lung cancer: a case report. Cureus.

[22] Lora DR, Post Z, Reau NS (2025). Immune checkpoint inhibitor-related hepatitis: beyond the guidelines.

[23] Miao K, Zhang L (2022). Incidence rate and treatment strategy of immune checkpoint inhibitor mediated hepatotoxicity: a systematic review. Cancer Pathog Ther.

[24] Farshidpour M, Hutson W (2022). Immune checkpoint inhibitors induced hepatotoxicity; gastroenterologists’ perspectives. Middle East J Dig Dis.

[25] Shojaie L, Ali M, Iorga A, Dara L (2021). Mechanisms of immune checkpoint inhibitor-mediated liver injury. Acta Pharm Sin B.

[26] Ferris RL, Blumenschein G, Fayette J (2016). Nivolumab for recurrent squamous-cell carcinoma of the head and neck. N Engl J Med.

[27] Kang YK, Boku N, Satoh T (2017). Nivolumab in patients with advanced gastric or gastro-oesophageal junction cancer refractory to, or intolerant of, at least two previous chemotherapy regimens (ONO-4538-12, ATTRACTION-2): a randomised, double-blind, placebo-controlled, phase 3 trial. Lancet.

[28] Satoh T, Kang YK, Chao Y (2020). Exploratory subgroup analysis of patients with prior trastuzumab use in the ATTRACTION-2 trial: a randomized phase III clinical trial investigating the efficacy and safety of nivolumab in patients with advanced gastric/gastroesophageal junction cancer. Gastric Cancer.

[29] Kelly RJ, Ajani JA, Kuzdzal J (2021). Adjuvant nivolumab in resected esophageal or gastroesophageal junction cancer. N Engl J Med.

[30] Larkin J, Minor D, D’Angelo S (2018). Overall survival in patients with advanced melanoma who received nivolumab versus investigator’s choice chemotherapy in CheckMate 037: a randomized, controlled, open-label phase III trial. J Clin Oncol.

[31] Spigel DR, Vicente D, Ciuleanu TE (2021). Second-line nivolumab in relapsed small-cell lung cancer: CheckMate 331^⋆^. Ann Oncol.

[32] André T, Shiu KK, Kim TW (2020). Pembrolizumab in microsatellite-instability-high advanced colorectal cancer. N Engl J Med.

[33] Chong WQ, Low JL, Tay JK (2025). Pembrolizumab with or without bevacizumab in platinum-resistant recurrent or metastatic nasopharyngeal carcinoma: a randomised, open-label, phase 2 trial. Lancet Oncol.

[34] Cortes J, Cescon DW, Rugo HS (2020). Pembrolizumab plus chemotherapy versus placebo plus chemotherapy for previously untreated locally recurrent inoperable or metastatic triple-negative breast cancer (KEYNOTE-355): a randomised, placebo-controlled, double-blind, phase 3 clinical trial. Lancet.

[35] Eggermont AMM, Blank CU, Mandala M (2018). Adjuvant pembrolizumab versus placebo in resected stage III melanoma. N Engl J Med.

[36] Finn RS, Ryoo BY, Merle P (2020). Pembrolizumab as second-line therapy in patients with advanced hepatocellular carcinoma in KEYNOTE-240: a randomized, double-blind, phase III trial. J Clin Oncol.

[37] Galsky MD, Mortazavi A, Milowsky MI (2020). Randomized double-blind phase II study of maintenance pembrolizumab versus placebo after first-line chemotherapy in patients with metastatic urothelial cancer. J Clin Oncol.

[38] Kudo M, Ren Z, Guo Y (2025). Transarterial chemoembolisation combined with lenvatinib plus pembrolizumab versus dual placebo for unresectable, non-metastatic hepatocellular carcinoma (LEAP-012): a multicentre, randomised, double-blind, phase 3 study. Lancet.

[39] Kuruvilla J, Ramchandren R, Santoro A (2021). Pembrolizumab versus brentuximab vedotin in relapsed or refractory classical Hodgkin lymphoma (KEYNOTE-204): an interim analysis of a multicentre, randomised, open-label, phase 3 study. Lancet Oncol.

[40] Langer CJ, Gadgeel SM, Borghaei H (2016). Carboplatin and pemetrexed with or without pembrolizumab for advanced, non-squamous non-small-cell lung cancer: a randomised, phase 2 cohort of the open-label KEYNOTE-021 study. Lancet Oncol.

[41] Rini BI, Plimack ER, Stus V (2019). Pembrolizumab plus axitinib versus sunitinib for advanced renal-cell carcinoma. N Engl J Med.

[42] Schmid P, Cortes J, Pusztai L (2020). Pembrolizumab for early triple-negative breast cancer. N Engl J Med.

[43] Ascierto PA, Stroyakovskiy D, Gogas H (2023). Overall survival with first-line atezolizumab in combination with vemurafenib and cobimetinib in BRAF(V600) mutation-positive advanced melanoma (IMspire150): second interim analysis of a multicentre, randomised, phase 3 study. Lancet Oncol.

[44] Bamias A, Davis ID, Galsky MD (2024). Atezolizumab monotherapy versus chemotherapy in untreated locally advanced or metastatic urothelial carcinoma (IMvigor130): final overall survival analysis from a randomised, controlled, phase 3 study. Lancet Oncol.

[45] Emens LA, Esteva FJ, Beresford M (2020). Trastuzumab emtansine plus atezolizumab versus trastuzumab emtansine plus placebo in previously treated, HER2-positive advanced breast cancer (KATE2): a phase 2, multicentre, randomised, double-blind trial. Lancet Oncol.

[46] Finn RS, Qin S, Ikeda M (2020). Atezolizumab plus bevacizumab in unresectable hepatocellular carcinoma. N Engl J Med.

[47] Grande E, Arranz J, De Santis M (2024). Atezolizumab plus chemotherapy versus placebo plus chemotherapy in untreated locally advanced or metastatic urothelial carcinoma (IMvigor130): final overall survival analysis results from a randomised, controlled, phase 3 study. Lancet Oncol.

[48] Gutzmer R, Stroyakovskiy D, Gogas H (2020). Atezolizumab, vemurafenib, and cobimetinib as first-line treatment for unresectable advanced BRAF(V600) mutation-positive melanoma (IMspire150): primary analysis of the randomised, double-blind, placebo-controlled, phase 3 trial. Lancet.

[49] Hida T, Kaji R, Satouchi M (2018). Atezolizumab in Japanese patients with previously treated advanced non-small-cell lung cancer: a subgroup analysis of the phase 3 OAK study. Clin Lung Cancer.

[50] Kelley RK, Rimassa L, Cheng AL (2022). Cabozantinib plus atezolizumab versus sorafenib for advanced hepatocellular carcinoma (COSMIC-312): a multicentre, open-label, randomised, phase 3 trial. Lancet Oncol.

[51] Kim S, Ghiringhelli F, de la Fouchardière C (2024). Atezolizumab plus modified docetaxel, cisplatin, and fluorouracil as first-line treatment for advanced anal cancer (SCARCE C17-02 PRODIGE 60): a randomised, non-comparative, phase 2 study. Lancet Oncol.

[52] Mansfield AS, Każarnowicz A, Karaseva N (2020). Safety and patient-reported outcomes of atezolizumab, carboplatin, and etoposide in extensive-stage small-cell lung cancer (IMpower133): a randomized phase I/III trial. Ann Oncol.

[53] Mittendorf EA, Zhang H, Barrios CH (2020). Neoadjuvant atezolizumab in combination with sequential nab-paclitaxel and anthracycline-based chemotherapy versus placebo and chemotherapy in patients with early-stage triple-negative breast cancer (IMpassion031): a randomised, double-blind, phase 3 trial. Lancet.

[54] Schmid P, Rugo HS, Adams S (2020). Atezolizumab plus nab-paclitaxel as first-line treatment for unresectable, locally advanced or metastatic triple-negative breast cancer (IMpassion130): updated efficacy results from a randomised, double-blind, placebo-controlled, phase 3 trial. Lancet Oncol.

[55] Sangro B, Kudo M, Erinjeri JP (2025). Durvalumab with or without bevacizumab with transarterial chemoembolisation in hepatocellular carcinoma (EMERALD-1): a multiregional, randomised, double-blind, placebo-controlled, phase 3 study. Lancet.

[56] Bang YJ, Ruiz EY, Van Cutsem E (2018). Phase III, randomised trial of avelumab versus physician’s choice of chemotherapy as third-line treatment of patients with advanced gastric or gastro-oesophageal junction cancer: Primary analysis of JAVELIN Gastric 300. Ann Oncol.

[57] Lee NY, Ferris RL, Psyrri A (2021). Avelumab plus standard-of-care chemoradiotherapy versus chemoradiotherapy alone in patients with locally advanced squamous cell carcinoma of the head and neck: a randomised, double-blind, placebo-controlled, multicentre, phase 3 trial. Lancet Oncol.

[58] Motzer RJ, Penkov K, Haanen J (2019). Avelumab plus axitinib versus sunitinib for advanced renal-cell carcinoma. N Engl J Med.

[59] Eggermont AM, Chiarion-Sileni V, Grob JJ (2015). Adjuvant ipilimumab versus placebo after complete resection of high-risk stage III melanoma (EORTC 18071): a randomised, double-blind, phase 3 trial. Lancet Oncol.

[60] Hodi FS, O’Day SJ, McDermott DF (2010). Improved survival with ipilimumab in patients with metastatic melanoma. N Engl J Med.

[61] Lynch TJ, Bondarenko I, Luft A (2012). Ipilimumab in combination with paclitaxel and carboplatin as first-line treatment in stage IIIB/IV non-small-cell lung cancer: results from a randomized, double-blind, multicenter phase II study. J Clin Oncol.

[62] Robert C, Thomas L, Bondarenko I (2011). Ipilimumab plus dacarbazine for previously untreated metastatic melanoma. N Engl J Med.

[63] Maio M, Scherpereel A, Calabrò L (2017). Tremelimumab as second-line or third-line treatment in relapsed malignant mesothelioma (DETERMINE): a multicentre, international, randomised, double-blind, placebo-controlled phase 2b trial. Lancet Oncol.

[64] Janjigian YY, Bendell J, Calvo E (2018). CheckMate-032 study: efficacy and safety of nivolumab and nivolumab plus ipilimumab in patients with metastatic esophagogastric cancer. J Clin Oncol.

[65] Larkin J, Chiarion-Sileni V, Gonzalez R (2015). Combined nivolumab and ipilimumab or monotherapy in untreated melanoma. N Engl J Med.

[66] Miller ED, Abu-Sbeih H, Styskel B (2020). Clinical characteristics and adverse impact of hepatotoxicity due to immune checkpoint inhibitors. Am J Gastroenterol.

[67] Postow MA, Chesney J, Pavlick AC (2015). Nivolumab and ipilimumab versus ipilimumab in untreated melanoma. N Engl J Med.

[68] Wang W, Lie P, Guo M, He J (2017). Risk of hepatotoxicity in cancer patients treated with immune checkpoint inhibitors: a systematic review and meta-analysis of published data. Int J Cancer.

[69] Fu J, Li WZ, McGrath NA (2021). Immune checkpoint inhibitor associated hepatotoxicity in primary liver cancer versus other cancers: a systematic review and meta-analysis. Front Oncol.

[70] Ascierto PA, Del Vecchio M, Robert C (2017). Ipilimumab 10 mg/kg versus ipilimumab 3 mg/kg in patients with unresectable or metastatic melanoma: a randomised, double-blind, multicentre, phase 3 trial. Lancet Oncol.

[71] Wang PF, Chen Y, Song SY (2017). Immune-related adverse events associated with anti-PD-1/PD-L1 treatment for malignancies: a meta-analysis. Front Pharmacol.

[72] Borghaei H, Paz-Ares L, Horn L (2015). Nivolumab versus docetaxel in advanced nonsquamous non-small-cell lung cancer. N Engl J Med.

[73] El-Khoueiry AB, Sangro B, Yau T (2017). Nivolumab in patients with advanced hepatocellular carcinoma (CheckMate 040): an open-label, non-comparative, phase 1/2 dose escalation and expansion trial. Lancet.

[74] Rizvi NA, Mazières J, Planchard D (2015). Activity and safety of nivolumab, an anti-PD-1 immune checkpoint inhibitor, for patients with advanced, refractory squamous non-small-cell lung cancer (CheckMate 063): a phase 2, single-arm trial. Lancet Oncol.

[75] Topalian SL, Sznol M, McDermott DF (2014). Survival, durable tumor remission, and long-term safety in patients with advanced melanoma receiving nivolumab. J Clin Oncol.

[76] Weber JS, D’Angelo SP, Minor D (2015). Nivolumab versus chemotherapy in patients with advanced melanoma who progressed after anti-CTLA-4 treatment (CheckMate 037): a randomised, controlled, open-label, phase 3 trial. Lancet Oncol.

[77] Robert C, Long GV, Brady B (2015). Nivolumab in previously untreated melanoma without BRAF mutation. N Engl J Med.

[78] Atallah E, Welsh SJ, O’Carrigan B (2023). Incidence, risk factors and outcomes of checkpoint inhibitor-induced liver injury: a 10-year real-world retrospective cohort study. JHEP Rep.

[79] Miah A, Tinoco G, Zhao S (2023). Immune checkpoint inhibitor-induced hepatitis injury: risk factors, outcomes, and impact on survival. J Cancer Res Clin Oncol.

[80] Pan J, Liu Y, Guo X (2022). Risk factors for immune-mediated hepatotoxicity in patients with cancer treated with immune checkpoint inhibitors: a systematic review and meta-analysis. Expert Opin Drug Saf.

[81] (2014). Keytruda FDA approval.

[82] Sprinzl MF, Galle PR (2013). Facing the dawn of immunotherapy for hepatocellular carcinoma. J Hepatol.

[83] Ravi S, Spencer K, Ruisi M (2014). Ipilimumab administration for advanced melanoma in patients with pre-existing hepatitis B or C infection: a multicenter, retrospective case series. J Immunother Cancer.

[84] Sangro B, Gomez-Martin C, de la Mata M (2013). A clinical trial of CTLA-4 blockade with tremelimumab in patients with hepatocellular carcinoma and chronic hepatitis C. J Hepatol.

[85] Ali FS, Nguyen MH, Hernaez R (2025). AGA clinical practice guideline on the prevention and treatment of hepatitis B virus reactivation in at-risk individuals. Gastroenterology.

[86] Yibirin M, Mustafayev K, Hosry J (2023). Immune checkpoint inhibitors suppress hepatitis C virus replication in infected patients with solid tumors. Am J Gastroenterol.

[87] Kumar V, Shinagare AB, Rennke HG (2020). The safety and efficacy of checkpoint inhibitors in transplant recipients: a case series and systematic review of literature. Oncologist.

[88] Biondani P, De Martin E, Samuel D (2018). Safety of an anti-PD-1 immune checkpoint inhibitor in a liver transplant recipient. Ann Oncol.

[89] Maggiore U, Pascual J (2016). The bad and the good news on cancer immunotherapy: implications for organ transplant recipients. Adv Chronic Kidney Dis.

[90] Varkaris A, Lewis DW, Nugent FW (2017). Preserved liver transplant after PD-1 pathway inhibitor for hepatocellular carcinoma. Am J Gastroenterol.

[91] Abdel-Wahab N, Shah M, Lopez-Olivo MA, Suarez-Almazor ME (2018). Use of immune checkpoint inhibitors in the treatment of patients with cancer and preexisting autoimmune disease: a systematic review. Ann Intern Med.

[92] Danlos FX, Voisin AL, Dyevre V (2018). Safety and efficacy of anti-programmed death 1 antibodies in patients with cancer and pre-existing autoimmune or inflammatory disease. Eur J Cancer.

[93] Kähler KC, Eigentler TK, Gesierich A (2018). Ipilimumab in metastatic melanoma patients with pre-existing autoimmune disorders. Cancer Immunol Immunother.

[94] Menzies AM, Johnson DB, Ramanujam S (2017). Anti-PD-1 therapy in patients with advanced melanoma and preexisting autoimmune disorders or major toxicity with ipilimumab. Ann Oncol.

[95] Tison A, Quéré G, Misery L (2019). Safety and efficacy of immune checkpoint inhibitors in patients with cancer and preexisting autoimmune disease: a nationwide, multicenter cohort study. Arthritis Rheumatol.

[96] Johnson DB, Sullivan RJ, Ott PA (2016). Ipilimumab therapy in patients with advanced melanoma and preexisting autoimmune disorders. JAMA Oncol.

[97] Björnsson HK, Björnsson ES (2022). Drug-induced liver injury: pathogenesis, epidemiology, clinical features, and practical management. Eur J Intern Med.

[98] Ye H, Nelson LJ, Gómez Del Moral M, Martínez-Naves E, Cubero FJ (2018). Dissecting the molecular pathophysiology of drug-induced liver injury. World J Gastroenterol.

[99] Fontana RJ (2014). Pathogenesis of idiosyncratic drug-induced liver injury and clinical perspectives. Gastroenterology.

[100] McKenzie J, Sneath E, Trinh A, Nolan M, Spain L (2024). Updates in the pathogenesis and management of immune-related enterocolitis, hepatitis and cardiovascular toxicities. Immunooncol Technol.

[101] Suzman DL, Pelosof L, Rosenberg A, Avigan MI (2018). Hepatotoxicity of immune checkpoint inhibitors: an evolving picture of risk associated with a vital class of immunotherapy agents. Liver Int.

[102] Alessandrino F, Tirumani SH, Krajewski KM (2017). Imaging of hepatic toxicity of systemic therapy in a tertiary cancer centre: chemotherapy, haematopoietic stem cell transplantation, molecular targeted therapies, and immune checkpoint inhibitors. Clin Radiol.

[103] De Martin E, Michot JM, Papouin B (2018). Characterization of liver injury induced by cancer immunotherapy using immune checkpoint inhibitors. J Hepatol.

[104] Riveiro-Barciela M, Barreira-Díaz A, Vidal-González J (2020). Immune-related hepatitis related to checkpoint inhibitors: clinical and prognostic factors. Liver Int.

[105] Weber JS, Kähler KC, Hauschild A (2012). Management of immune-related adverse events and kinetics of response with ipilimumab. J Clin Oncol.

[106] Huffman BM, Kottschade LA, Kamath PS, Markovic SN (2018). Hepatotoxicity after immune checkpoint inhibitor therapy in melanoma: natural progression and management. Am J Clin Oncol.

[107] U.S. Department of Health and Human Services (2017). Common Terminology Criteria for Adverse Events (CTCAE) version 5.0.

[108] Fontana RJ, Watkins PB, Bonkovsky HL (2009). Drug-Induced Liver Injury Network (DILIN) prospective study: rationale, design and conduct. Drug Saf.

[109] Bolte FJ, Hall RD, Shah NL (2022). Immune checkpoint inhibitor-related liver toxicity. Clin Liver Dis (Hoboken).

[110] Berry P, Kotha S, Zen Y (2023). Immune checkpoint inhibitor-related cholangiopathy: novel clinicopathological description of a multi-centre cohort. Liver Int.

[111] Moi L, Bouchaab H, Mederos N (2021). Personalized cytokine-directed therapy with tocilizumab for refractory immune checkpoint inhibitor-related cholangiohepatitis. J Thorac Oncol.

[112] Onoyama T, Takeda Y, Yamashita T (2020). Programmed cell death-1 inhibitor-related sclerosing cholangitis: a systematic review. World J Gastroenterol.

[113] Sawada K, Shonaka T, Nishikawa Y (2019). Successful treatment of nivolumab-related cholangitis with prednisolone: a case report and review of the literature. Intern Med.

[114] Zhuang D, Zhang D, Riordan S (2024). Hepatobiliary complications of immune checkpoint inhibitors in cancer. Explor Target Antitumor Ther.

[115] Bessone F, Bjornsson ES (2022). Checkpoint inhibitor-induced hepatotoxicity: role of liver biopsy and management approach. World J Hepatol.

[116] Dougan M, Wang Y, Rubio-Tapia A, Lim JK (2021). AGA clinical practice update on diagnosis and management of immune checkpoint inhibitor colitis and hepatitis: expert review. Gastroenterology.

[117] Li M, Sack JS, Bell P (2021). Utility of liver biopsy in diagnosis and management of high-grade immune checkpoint inhibitor hepatitis in patients with cancer. JAMA Oncol.

[118] Parlati L, Marcin K, Terris B (2023). Histological characteristics and management of hepatitis on immune checkpoint inhibitors: a retrospective descriptive study. J Clin Med.

[119] Schneider BJ, Naidoo J, Santomasso BD (2021). Management of immune-related adverse events in patients treated with immune checkpoint inhibitor therapy: ASCO guideline update. J Clin Oncol.

[120] Xie X, He X, Ye X (2024). Clinical significance of liver biopsy in the diagnosis of liver disease and the evaluation of the clinical efficacy of antiviral treatment for chronic hepatitis B. Am J Transl Res.

[121] Escobar D (2021). Pathology pearls: immune checkpoint inhibitor hepatitis.

[122] Ito T, Takeuchi Y, Mizuno K (2024). Diagnostic guide for immune checkpoint inhibitor-induced liver injury. Hepatol Res.

[123] Haanen J, Obeid M, Spain L (2022). Management of toxicities from immunotherapy: ESMO clinical practice guideline for diagnosis, treatment and follow-up. Ann Oncol.

[124] Dougan M, Blidner AG, Choi J (2020). Multinational Association of Supportive Care in Cancer (MASCC) 2020 clinical practice recommendations for the man-agement of severe gastrointestinal and hepatic toxicities from checkpoint inhibitors. Support Care Cancer.

[125] National Comprehensive Cancer Network (2024). Management of immunotherapy-related toxicities. Version 1.2024. NCCN Clinical Practice Guidelines in Oncology.

[126] Brahmer JR, Abu-Sbeih H, Ascierto PA (2021). Society for Immunotherapy of Cancer (SITC) clinical practice guideline on immune checkpoint inhibitor-related adverse events. J Immunother Cancer.

