# Supplementary material for: Immune Checkpoint Inhibitor-induced Hepatitis, an Emerging Issue in Precision Cancer Therapy Era: A Narrative Literature Review
Source: Rambam Maimonides Med J. 2026 Jan 28;17(1):e0005. doi: 10.5041/RMMJ.10571 (PMC12857653; doi:10.5041/RMMJ.10571)
Supplement: Supplementary file 1 [file rmmj-17-1-e0005_supplement.docx]

This appendix has been provided by the authors for the benefit of readers

Supplement to Immune Checkpoint Inhibitor-induced Hepatitis, an Emerging Issue in Precision Cancer Therapy Era: A Narrative Literature Review

Adiwinata R, Tanadi C, Pajala BF, Tandarto K, Stella MM, Tenggara JB, Bunarsa RG, Simadibrata P, Siregar L, Nababan SHH, Sulaiman BS, Hasan I, Lesmana CRA, Sudoyo AW. Immune Checkpoint Inhibitor-induced Hepatitis, an Emerging Issue in Precision Cancer Therapy Era: A Narrative Literature Review. Rambam Maimonides Med J 2026;17 (1):e0005. Review. doi:10.5041/RMMJ.10571

Supplementary Table 1. Comparison of the Management of ILICI in Different Guidelines.^1-8^

| Grade | AASLD | AGA | ASCO | EASL | ESMO | MASCC | NCCN | SITC |
| --- | --- | --- | --- | --- | --- | --- | --- | --- |
| Grade 1 | 1. Continue ICI 2. Monitor closely 3. Investigate other causes of hepatitis | 1. Continue ICI if asymptomatic 2. Monitor closely 3. Investigate other causes of hepatitis | 1. Continue ICI if asymptomatic 2. Monitor closely 3. Investigate other causes of hepatitis 4. Supportive care | 1. Continue ICI if irAEs are excluded (unlikely or untreated) 2. Monitor closely 3. Investigate possible cause of hepatitis 4. Symptomatic treatment | 1. Continue ICI 2. Monitor liver parameters | 1. Stop potentially hepatotoxic concomitant medications and supplements 2. Advise against alcohol use 3. Monitor liver parameters once to twice weekly | 1. Consider withholding ICI 2. Monitor liver parameters 3. Investigate causes of hepatitis | 1. Continue ICI 2. Monitor liver parameters weekly 3. Investigate causes of hepatitis |
| Grade 2 | 1. Withhold ICI temporarily 2. Oral prednisone 0.5-1.0 mg/kg/day | 1. Withhold ICI temporarily 2. Oral prednisone 0.5-1.0 mg/kg/ day and taper ≥1 month if condi­tion improves 3. Consider liver biopsy 4. Resume ICI if condition improves to grade ≤1 hepatitis | 1. Withhold ICI temporarily 2. Administer oral prednisone 0.5-1.0 mg/kg/day if no improvement after 3 days and taper ≥1 month if condi­tion improves 3. Withhold other potentially hepatotoxic drugs 4. Monitor closely 5. Consider liver biopsy for per­sistently elevated liver tests 6. Consider adding mycophen­olate mofetil if no improvement after 3 days 7. Resume ICI if con­dition improves to grade ≤1 hepa­titis on prednisone ≤10 mg/day | 1. Withhold ICI 2. Monitor liver parameters, INR and albumin twice weekly 3. If abnormal liver parameters persist >2 weeks start immuno­suppression and discontinue drug 4. ICI may be restarted after steroid taper | 1. Withhold ICI 2. Start oral prednisolone 1 mg/kg/day if rising ALT/AST when re-checked 3. Monitor liver parameters, INR and albumin every 3 days 4. Investigate other causes of hepatitis 5. ICI may be resumed once prednisone ≤10 mg | 1. Withhold ICI temporarily 2. Oral pred­nisolone 0.5-1.0 mg/kg/day 3. Twice weekly liver testing 4. Investigate other causes of hepatitis 5. Consider liver biopsy 6. Add azathio­prine 1-2 mg/kg daily or myco­phenolate mofetil 500-1000 mg twice a day, or tacro­limus 8-10 ng/mL or lower if refractory to steroid 7. ICI may be resumed when condition im­proved to grade ≤1 hepatitis | 1. Withhold ICI 2. Consider prednisone 0.5-1.0 mg/kg/day 3. Monitor liver parameters every 3-5 days 4. Monitor PT/INR periodically 5. Check creatinine phosphokinase and aldolase to rule out myositis 6. ICI may be resumed when condition improved to grade ≤1 hepatitis | 1. Withhold ICI 2. Prednisone 0.5-1.0 mg/kg/day with 4-week taper 3. Monitor liver parameters weekly 4. Investigate other causes of hepatitis 5. Consider liver biopsy 6. Restart ICI when steroid taper to 10 mg/day (tox­icity grade ≤1) |
| Grade 3 | 1. Permanently discontinue ICI 2. IV steroid 1-1.5 mg/kg/day 3. Liver tests every 2-4 weeks after tapering of immuno-suppression 4. Hospitalize patients with jaundice 5. Mycophenolate mofetil or azathioprine for those refractory to steroid | 1. Permanently discontinue ICI 2. IV methylpred­nisolone 1-2 mg/kg/day and taper ≥1 month if condition improves 3. Consider hospitalization 4. Consider liver biopsy 5. Mycophenolate mofetil, tacrolimus, or azathioprine for those refractory to steroid 6. Consider antithymocyte globulin if fulminant | 1. Permanently discontinue ICI if symptomatic 2. IV methylpred­nisolone 1-2 mg/kg/day and taper over 4-6 weeks when grade ≤1 hepatitis 3. Consider hos­pitalization and transfer to terti­ary care facility if indicated 4. Consider liver biopsy if steroid-refractory 5. Consider add­ing mycophen­olate mofetil or azathio­prine. Tac­rolimus is reserved for those refrac­tory to mycophen­olate mofetil 6. Refer to hepa­tologist if no im­provement after receiving steroid or for patients with ICI combined with novel agent, with standard chemotherapy, or with targeted therapy | 1. Consider permanently discontinuing ICI 2. Start methyl­prednisolone 1-2 mg/kg/day 3. Monitor liver parameters and INR daily 4. Hospital admis­sion if impend­ing liver failure (bilirubin ≥2.5 mg/dL and/or INR ≥1.5) 5. Consider liver biopsy 6. Add mycopheno­late mofetil 1000 mg twice daily if no re­sponse to ste­roids after 2-3 days 7. Add additional immunosuppres­sion (mycophe­no­late mofetil, cyclosporine, tacrolimus, anti-thymocyte glob­u­lin) if refrac­tory to steroid 8. Withdraw hepa­totoxic drugs | 1. Discontinue ICI 2. If ALT/AST <400 and normal bilirubin/INR/albu-min, start oral prednisolone 1 mg/kg/day. Otherwise, start methylprednisolone 2 mg/kg/day IV 3. Monitor liver parameters, INR, albumin daily 4. Perform ultrasound with Doppler 5. Consider hospitalization if the condition is clinically concerning 6. If refractory to steroid, add mycophenolate mofetil 500-1000 mg once a day IV or tacrolimus or triple therapy | 1. Same as grade 2 2. Discontinue ICI 3. Prednisone 0.5-2.0 mg/kg twice a day 4. Daily to every other day liver tests 5. Liver biopsy 6. Triple therapy may be considered in refractory cases | 1. Withhold ICI 2. IV methylpred­nisolone 1 mg/kg/day 3. Monitor liver parameters every 1-5 days 4. Monitor PT/INR periodically 5. Consider hospitalization if synthetic hepatic dysfunction is observed 6. Consider liver biopsy 7. Add steroid-sparing immunosuppressive therapy (mycophenolate mofetil, tacrolimus, azathioprine, tocilizumab) if no improvement with steroids for 1-2 days | 1. Permanently discontinue ICI 2. Prednisone 1-2 mg/kg/day 3. Monitor liver parameters every 1-2 days 4. Consider liver biopsy, MRI, and CT scan 5. Consider mycophenolate mofetil if refractory to steroid after 3 days 6. If AST/ALT did not improve to grade 1 within 10-14 days of steroid initia­tion, or if liver toxicity recurs after steroid taper, mycophe­nolate mofetil, tacrolimus, or antithymocyte globulin may be considered |
| Grade 4 | Same as grade 3 | Same as grade 3 | 1. Same as grade 3 2. IV methylpred­nisolone 2 mg/kg/ day | Same as grade 3 | 1. Same as grade 3 2. Permanently discontinue ICI 3. IV methylpred­nisolone 2 mg/kg/ day 4. Consider liver biopsy 5. Consult hepatology | 1. Same as grade 3 2. Daily liver tests 3. Check acetamin­ophen level 4. Consider hos­pitalization for IV steroids and close monitoring 5. Antithymocyte globulin may be con­sidered in rapidly progressing hepatitis | 1. Same as grade 3 2. Monitor liver parameters every 1-3 days 3. Hospitalize patient | Same as grade 3 |

AASLD: American Association for the Study of Liver Disease; AGA, American Gastroenterology Association; ALT, alanine transaminase; ASCO, American Society of Clinical Oncology; AST, aspartate aminotransferase; EASL, European Association for the Study of the Liver; ESMO, European Society for Medical Oncology; ICI, immune checkpoint inhibitor; ILICI, immune-mediated liver injury caused by ICIs; irAEs, ICI immune-related adverse events; INR, international normalized ratio; IV, intravenous; kg, kilogram; MASCC, Multinational Association of Supportive Care in Cancer; mg, milligram; mL, milliliter; NCCN, National Comprehensive Cancer Network; PT, prothrombin; SITC, Society for Immunotherapy of Cancer.

# References

1. Fontana RJ, Liou I, Reuben A, et al. AASLD practice guidance on drug, herbal, and dietary supplement-induced liver injury. Hepatology 2023;77:1036–65. <https://doi.org/10.1002/hep.32689>
2. Dougan M, Wang Y, Rubio-Tapia A, Lim JK. AGA clinical practice update on diagnosis and management of immune checkpoint inhibitor colitis and hepatitis: expert review. Gastroenterology 2021;160:1384–93. <https://doi.org/10.1053/j.gastro.2020.08.063>
3. Schneider BJ, Naidoo J, Santomasso BD, et al. Management of immune-related adverse events in patients treated with immune checkpoint inhibitor therapy: ASCO guideline update. J Clin Oncol 2021;39:4073–126. <https://doi.org/10.1200/JCO.21.01440>
4. European Association for the Study of the Liver. EASL clinical practice guidelines: drug-induced liver injury. J Hepatol 2019;70:1222–61. <https://doi.org/10.1016/j.jhep.2019.02.014>
5. Haanen J, Obeid M, Spain L, et al. Management of toxicities from immunotherapy: ESMO clinical practice guideline for diagnosis, treatment and follow-up. Ann Oncol 2022;33:1217–38. <https://doi.org/10.1016/j.annonc.2022.10.001>
6. Dougan M, Blidner AG, Choi J, et al. Multinational Association of Supportive Care in Cancer (MASCC) 2020 clinical practice recommendations for the management of severe gastrointestinal and hepatic toxicities from checkpoint inhibitors. Support Care Cancer 2020;28:6129–43. <https://doi.org/10.1007/s00520-020-05707-3>
7. National Comprehensive Cancer Network. Management of immunotherapy-related toxicities. Version 1.2024. NCCN Clinical Practice Guidelines in Oncology. National Comprehensive Cancer Network; 2024. Available at: <https://www.nccn.org/guidelines/guidelines-detail?category=3&id=1486> (accessed December 30, 2025).
8. Brahmer JR, Abu-Sbeih H, Ascierto PA, et al. Society for Immunotherapy of Cancer (SITC) clinical practice guideline on immune checkpoint inhibitor-related adverse events. J Immunother Cancer 2021;9:e002435. <https://doi.org/10.1136/jitc-2021-002435>
